# Fast dynamic voluntary contractions enhance corticospinal facilitation of the human diaphragm: A transcranial magnetic stimulation study

**DOI:** 10.14814/phy2.70905

**Published:** 2026-05-05

**Authors:** Alexandre Demoule, Eric Verin, Capucine Morélot‐Panzini, Christian Straus, Thomas Similowski

**Affiliations:** ^1^ Sorbonne Université, INSERM, UMRS1158 Neurophysiologie Respiratoire Expérimentale et Clinique Paris France; ^2^ Département R3S, Service de Médecine Intensive et Réanimation AP‐HP, Groupe Hospitalier Universitaire APHP‐Sorbonne Université, Hôpital Pitié‐Salpêtrière Paris France; ^3^ Service de Médecine de Réadaptation Respiratoire, Centre Hospitalo‐Universitaire de Rouen Rouen France; ^4^ Université Rouen‐Normandie, GRHVN UR 3838 Rouen France; ^5^ Service de Pneumologie, Département R3S AP‐HP, Groupe Hospitalier Universitaire APHP‐Sorbonne Université, Hôpital Pitié‐Salpêtrière Paris France; ^6^ Service des Explorations Fonctionnelles de la Respiration, de l'Exercice et de la Dyspnée, Département R3S AP‐HP, Groupe Hospitalier Universitaire APHP‐Sorbonne Université, Hôpital Pitié‐Salpêtrière Paris France; ^7^ Département R3S AP‐HP, Groupe Hospitalier Universitaire APHP‐Sorbonne Université, Hôpital Pitié‐Salpêtrière Paris France

**Keywords:** corticospinal excitability, diaphragm, dynamic contraction, motor control, motor evoked potentials, transcranial magnetic stimulation

## Abstract

In limb muscles, corticospinal excitability is modulated by motor context, with greater facilitation during movement initiation and dynamic contractions than during sustained isometric activation. Whether this applies to the human diaphragm remains uncertain, given the hybrid automatic‐voluntary control and continuous activity of respiratory motoneurones. To determine whether corticospinal excitability of the human diaphragm is influenced by the dynamics of voluntary inspiratory contraction at a given level of inspiratory mouth pressure, nine healthy participants (3 women, 6 men; age 23–39 years) performed inspiratory efforts against an occluded mouthpiece. Diaphragm motor evoked potentials (Di‐MEPs) elicited by transcranial magnetic stimulation were recorded from validated chest surface sites at end‐expiration with the airway occluded, at rest, during sustained static inspiratory efforts at graded fractions of maximal inspiratory mouth pressure (Pi,max), and during dynamic inspiratory efforts matched for pressure (20% Pi,max) but differing in rate of pressure development (slow vs. fast). Static efforts increased Di‐MEP amplitude and shortened latency in a pressure‐dependent manner. Slow dynamic efforts produced similar facilitation to static efforts. Fast dynamic efforts elicited greater facilitation, with increased amplitude and shortened latency. Corticospinal excitability of the diaphragm is modulated by contraction dynamics, with rapid efforts inducing additional facilitation beyond force alone.

## 1 INTRODUCTION

In limb muscles, corticospinal excitability is modulated by motor context. When assessed with transcranial magnetic stimulation (TMS), this manifests as enhanced responses during isometric contractions compared with rest (Hess et al., 1987), with further enhancement during movement initiation and dynamic or ballistic contractions (Aranyi et al., 1998; Chen et al., 1998; Ni et al., 2006). Increased motor evoked potential (MEP) amplitude and shortened latency attest to transient changes in corticospinal output (Bestmann & Krakauer, 2015; Rothwell, 1997). These state‐ and task‐related changes are integral to the flexible control of voluntary movement (Bestmann & Krakauer, 2015). This may differ for respiratory muscles, which operate outside the canonical voluntary motor system because of their dual automatic and voluntary control.

Indeed, respiratory motoneurones are continuously bombarded by multiple breathing‐related inputs (Aminoff & Sears, 1971), including the descending drive from automatic brainstem respiratory networks and many afferent messages (Feldman & Del Negro, 2006). In addition, they directly or indirectly receive non‐respiratory influences, such as emotion‐related signals and somatosensory or nociceptive inputs (Davenport & Vovk, 2009; Van Diest et al., 2009). The integration of these messages through a complex network of interneurones (Sunshine et al., 2020) results in fluctuations of motoneuronal membrane depolarisation, which may be facilitating or disfacilitating depending on context. While voluntary motor commands to respiratory muscles can transiently override the automatic ventilatory drive (McKay et al., 2003; Schottelkotte & Crone, 2022), they do so within this continuously active system. As a result, these commands may be processed differently from those directed to limb muscles, particularly during brief or rapidly changing contractions.

In this context, diaphragmatic responses to TMS have been studied primarily at rest (end‐expiration) or during steady voluntary contractions (Sharshar et al., 2003; Similowski et al., 1996). Under these conditions, the diaphragm behaves like a limb muscle, with MEP amplitude increasing and latency decreasing with background activation (Devanne et al., 1997; Kischka et al., 1993; Plassman & Gandevia, 1989). Two studies examined the influence of automatic breathing on corticodiaphragmatic responses (Mehiri et al., 2006; Straus et al., 2004), but the influence of the dynamics of voluntary diaphragmatic contraction has received little attention. As a result, it remains unclear whether the diaphragm shows the same transient facilitation during dynamic contractions as limb muscles, or whether its hybrid control organization modifies this relationship.

We hypothesized that, if the dynamic corticospinal modulation described in limb muscles extends to respiratory muscles, dynamic voluntary inspirations would elicit greater facilitation than static ones matched for mechanical output. Diaphragm MEPs (Di‐MEPs) were therefore compared during sustained and slow and fast dynamic inspiratory efforts performed at equivalent inspiratory pressures.

## 2 MATERIALS AND METHODS

### 2.1 Participants and ethical approval

Nine healthy volunteers were included (3 women, men; 23 to 39 years; body mass index 19–27.4 kg/m^2^) after completion of the French legal procedure for biomedical research. The study was approved by the “*Comité Consultatif de Protection des Personnes se prêtant à des Recherches Biomédicales, Pitié‐Salpêtrière*”. All participants were fully informed and provided written informed consent.

### 2.2 Measurements

#### 2.2.1 Electromyograms

Di‐MEPs were recorded using pairs of skin‐taped silver cup electrodes filled with conductive paste, positioned according to a previously described technique (Demoule et al., 2003) Di‐MEPs were retained for analysis when associated with a clear abdominal expansion attesting to a diaphragmatic contraction and in the absence of obvious electrical artifact or EKG contamination. Di‐MEPs' latencies were measured from stimulus onset to the first departure from baseline and expressed in absolute values (ms). Di‐MEPs' amplitudes were measured from peak to peak and expressed relative to resting baseline (percentage change).

#### 2.2.2 Respiratory pressure

Mouth pressure (Pm) was measured via a flanged mouthpiece connected to a linear differential pressure transducer (0–150 cmH_2_O; CD15‐C, Validyne, Northridge, CA, USA).

#### 2.2.3 Abdominal displacements

Changes in abdominal (AB) circumference were monitored using a mechanical strain gauge with a piezo‐electric sensor encased in a molded box (Nihon Kohden, Tokyo, Japan) attached to an elastic belt at the level of the umbilicus.

All signals were fed into a Nihon Kohden Neuropack Sigma® electromyograph (Tokyo, Japan), sampled at 10 kHz with a bandwidth of 20 Hz to 5 kHz, and stored on a portable computer for subsequent analysis (PowerLab®, AD Instruments, Hastings, UK).

### 2.3 Stimulations

All stimulations were performed using a Magstim 200 stimulator (Whitland, Dyfed, UK) equipped with a 90 mm circular coil (peak magnetic field 2.5 Tesla). Stimuli were delivered at end‐expiration identified on the abdominal displacement signal, against an occluded airway. For each condition, three to five consecutive stimulations were applied. TMS was delivered with the coil positioned over the vertex, the handle oriented in the sagittal plane. The anteroposterior coil position was optimized based on the diaphragmatic motor evoked potential and then kept constant.

### 2.4 Experimental sequence

In each participant, maximal static inspiratory mouth pressure (Pi,max) was first measured according to recommended procedures (Laveneziana et al., 2019).

The resting diaphragmatic motor threshold (Di‐Mthr) was then determined by decreasing the stimulator output by 5% steps from 100% to Di‐MEPs disappeareance. The lowest intensity eliciting a Di‐MEP in at least two out of three stimulations was retained as Di‐Mthr. Baseline Di‐MEP latency (from stimulus to first departure from baseline) and amplitude (peak‐to‐peak) were established at 105% of Di‐Mthr.

To characterize the facilitatory effects of static inspiratory efforts, participants were instructed to inspire against an occluded mouthpiece while simultaneously decreasing Pm and increasing AB (visual biofeedback) to ensure diaphragmatic contribution. TMS was delivered at the end of a 5‐s sustained inspiratory effort producing Pm corresponding to 20%, 40%, 60%, and 80% of Pi,max.

The facilitatory effects of dynamic inspiratory efforts, performed using the same instructions, were characterized during slow efforts, defined by a rate of pressure development below 15 cmH_2_O·s^−1^, and fast efforts, defined by a rate exceeding 50 cmH_2_O·s^−1^. The stimulator was triggered when Pm reached 20% of Pi,max.

### 2.5 Data analysis

#### 2.5.1 Statistics

Data analyses and visualizations were performed using R (R Foundation, Vienna, Austria; version 4.5.2) within the RStudio environment (Posit Software, Boston, MA, USA). Analyses were performed at the subject level after aggregation of repeated trials, with values summarized by their median for each subject and condition. Results are presented as medians with interquartile ranges [Q1–Q3]. Effect sizes were quantified as within‐subject differences. Given the small sample size and the absence of distributional assumptions, statistical analyses relied on paired nonparametric tests. Each condition was compared with resting values using two‐sided Wilcoxon signed‐rank tests, with Holm adjustment for multiple comparisons. In addition, planned pairwise comparisons between conditions were conducted using the same approach. Statistical significance was set at a two‐sided alpha level of 0.05.

## 3 RESULTS

Pi,max ranged from 86 to 105 cmH_2_O in men and from 73 to 90 cmH_2_O in women, within normal values (Laveneziana et al., 2019). In all participants under relaxation conditions, TMS at maximal stimulator output elicited a clear di‐MEP. Median MthR stimulation output was 85% [75;90] of maximum. Di‐MEP latency at 105% of MthR under resting condition was 17.3 ms [17;18.1].

**FIGURE 1:**
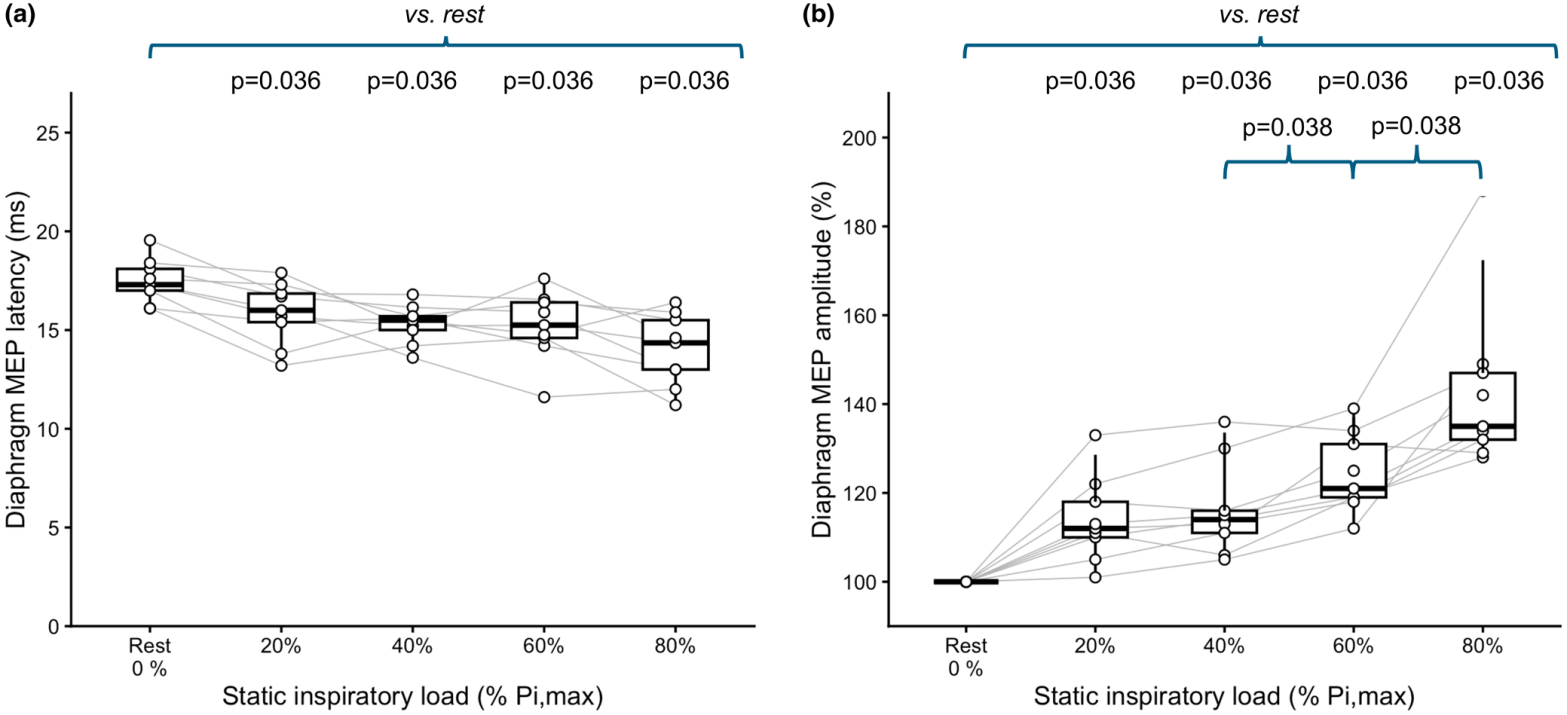
Effects of graded static inspiratory efforts on diaphragmatic motor evoked potential (Di‐MEP) latency (a) and amplitude (b) in response to transcranial magnetic stimulation. Boxplots represent medians and interquartile ranges, with indication of the 95th percentile of the distribution. Individual data are shown with paired trajectories. *p* Values after Holm's correction.

During static inspiratory efforts, MEP amplitude increased and latency decreased, with consistent effects across all levels. Latency shortening was observed in all participants and was significant at all levels (all *p* = 0.009, Holm‐adjusted *p* = 0.036) with progressive shortening from −1.4 ms [−2.7; −0.7] at 20% to −3.8 ms [−4.9; −2.95] at 80% Pi,max (Figure 1a). Amplitude facilitation was also observed in all participants and at all levels (all *p* = 0.009, Holm‐adjusted *p* = 0.036), to reach +35% [32–47] at 80% Pi,max (Figure 1b).

**FIGURE 2:**
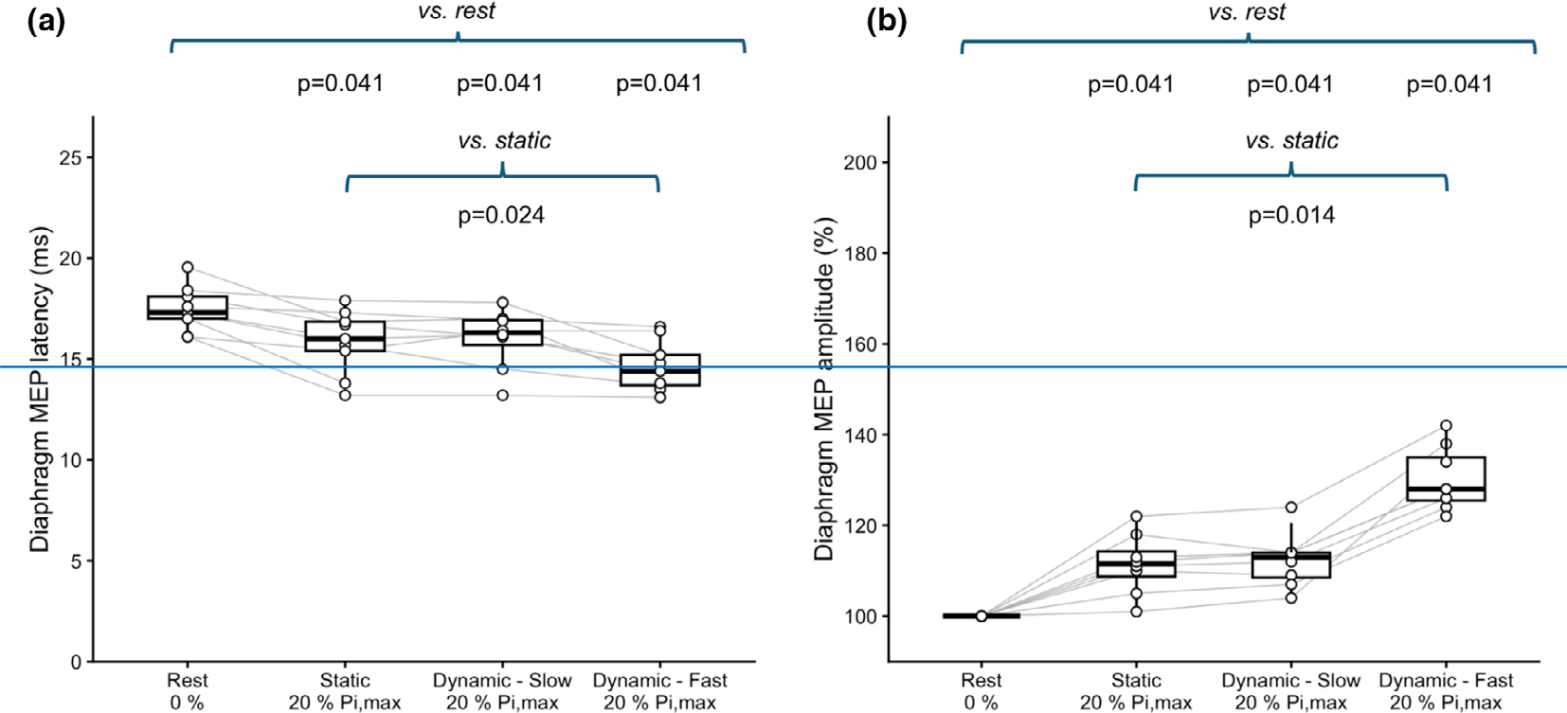
Effects of inspiratory efforts corresponding to 20% of maximal inspiratory pressure (Pi,max), performed under static, slow dynamic, and fast dynamic conditions, on diaphragmatic motor evoked potential (Di‐MEP) latency (a) and amplitude (b) in response to transcranial magnetic stimulation. Boxplots represent medians and interquartile ranges, with indication of the 95th percentile of the distribution. Individual data are shown with paired trajectories. *p* Values after Holm's correction.

Slow dynamic inspiratory efforts were performed at a Pm rate of rise between 6 and 9.4 cmH_2_O·s^−1^, versus 95–170 cmH_2_O·s^−1^ for fast efforts. At 20% Pi,max, slow dynamic inspiratory efforts produced MEP facilitation comparable to that observed during static loading (Figure 2). In contrast, fast dynamic inspiratory efforts induced a distinct additional modulation. Amplitude increased to 128% [126–135], corresponding to a further gain of +16.5% [15–20] compared with static loading (*p* = 0.014), while latency showed a greater shortening (*p* = 0.024). These effects were consistent across participants (Figure 2).

**FIGURE 3:**
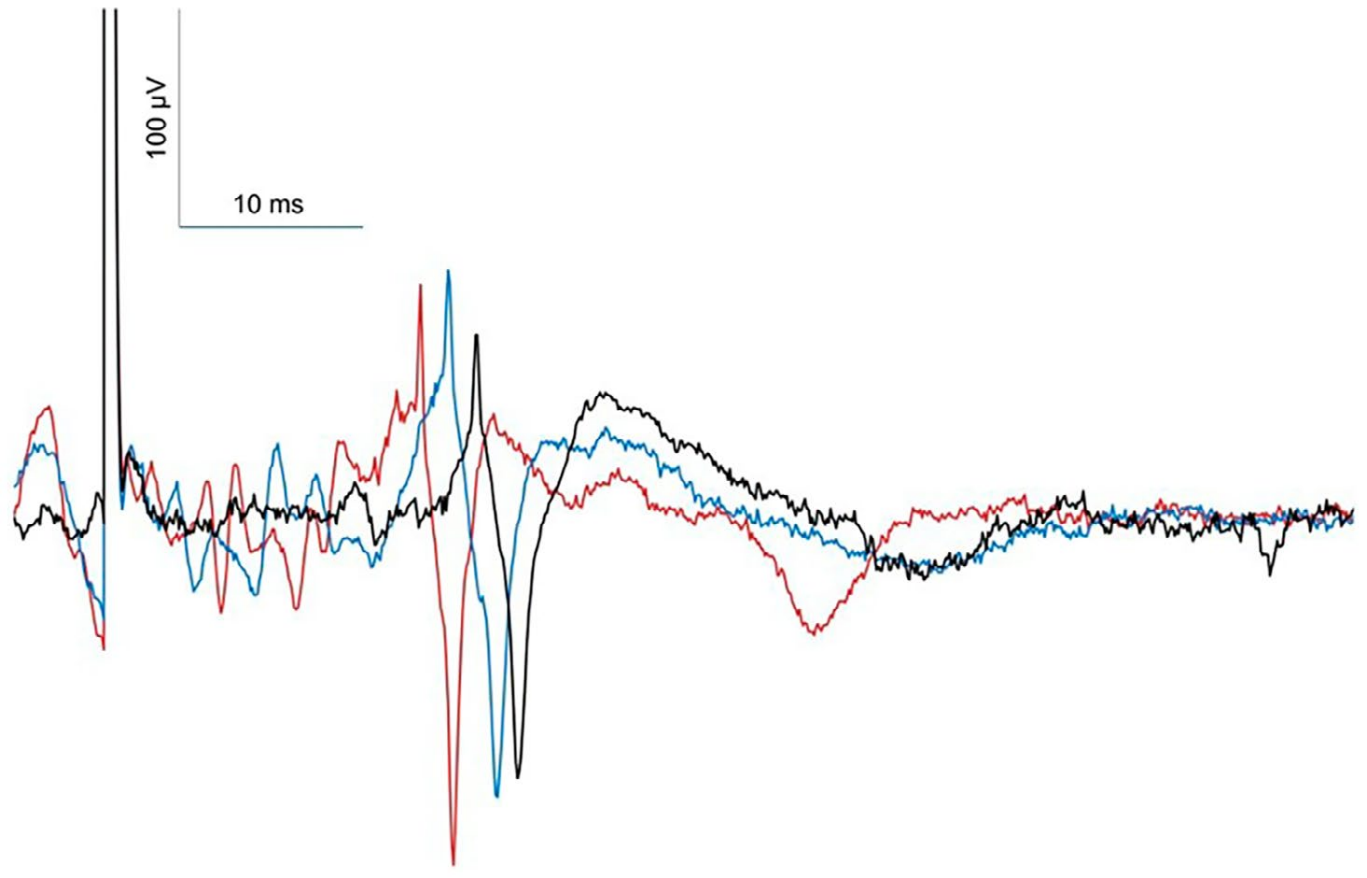
Diaphragm motor evoked potentials at rest (black trace), during a static contraction corresponding to 20% of maximal inspiratory pressure (blue trace), and during a fast dynamic contraction at the same level of inspiratory pressure (red trace), in a representative participant.

Figure 3 illustrates these results in one representative participant.

## 4 DISCUSSION

This study shows that diaphragmatic corticospinal excitability is not only modulated by the strength of a voluntary inspiratory activation but also by its dynamics. As expected (Sharshar et al., 2003; Similowski et al., 1996), increasing background inspiratory effort progressively increased Di‐MEP amplitude and shortened latency during static maneuvers. At the same level of mechanical output, fast dynamic inspiratory efforts elicited greater facilitation than sustained efforts. This mirrors the limb muscle pattern (Aranyi et al., 1998; Chen et al., 1998; Ni et al., 2006), extending it to the diaphragm.

Previous work showed that the automatic inspiratory drive is sufficient to dynamically modulate phrenic corticospinal excitability, as evidenced by inspiratory shortening of Di‐MEP latency during quiet breathing (Mehiri et al., 2006), or Di‐MEP facilitation during carbon dioxide stimulation (Murphy et al., 1990; Straus et al., 2004), with facilitation documented to occur the spinal level (Davey et al., 1996). Voluntary inspiratory also facilitate Di‐MEPs (Murphy et al., 1990; Similowski et al., 1996). The present study confirms that task‐dependent facilitation exists for phrenic motoneurones despite their particular operating environment and, additionally, evidences a particular pattern of response. Slow inspiratory efforts produced facilitation similar to sustained contractions, whereas fast voluntary inspiratory efforts induced an additional, transient facilitation. This contrasts with studies on the abductor pollicis brevis showing increased MEP amplitude during both slow and fast dynamic contractions (Aranyi et al., 1998; Chen et al., 1998; Ni et al., 2006). The slow–fast dissociation observed in the diaphragm suggests that the continuous automatic activity of respiratory motoneurones may already place them in a facilitated state in which slowly varying voluntary drive is largely integrated, leaving little room for further modulation. In contrast, rapid voluntary commands appear capable of recruiting supplementary facilitatory mechanisms.

Changes in Di‐MEP amplitude and latency closely paralleled one another across conditions. Such coupled modulation is consistent with state‐dependent changes in motoneuronal excitability and the synchronization of descending volleys, as described in limb muscles (Di Lazzaro et al., 1998; Rothwell et al., 1987). Notably, in contrast to published hand muscle observations (Devanne et al., 1997; Hess et al., 1987), Di‐MEP latency did not reach an early plateau but continued to shorten with increasing inspiratory effort and contraction speed at a given effort level. In distal hand muscles, latency shortening and MEP facilitation typically plateau at relatively low force levels, suggesting an early saturation of corticospinal and motoneuronal recruitment, and increases in MEP amplitude during dynamic contractions are not consistently accompanied by latency changes. Although our design does not allow precise localisation of the underlying mechanisms, the present pattern supports a genuine modulation of corticospinal transmission (Di Lazzaro et al., 1999; Taylor & Gandevia, 2004). The absence of an early plateau in latency shortening suggests that, in the diaphragm, this saturation may occur at higher levels of activation or may be less sharply defined, and that diaphragmatic motoneurones remain responsive to transient increases in excitability over a wide operating range.

Several limitations warrant consideration. The small sample size limits quantitative generalization, although within‐subject effects were consistent. Pm was used as the operational index of inspiratory effort rather than transdiaphragmatic pressure. Although noninvasive and clinically relevant, Pm reflects the integrated action of multiple inspiratory muscles, and the relative contribution of the diaphragm may differ between static and dynamic efforts. We consider the contribution of such differences to the observed effects unlikely because precautions were taken to ensure diaphragmatic participation in inspiratory efforts through monitoring of abdominal expansion (De Troyer & Estenne, 1988; Mead & Loring, 1982), but we acknowledge that this is not a perfect solution. Finally, MEPs were retained only when accompanied by clear abdominal displacement, and electrodes were carefully placed (Demoule et al., 2003), making crosstalk from abdominal muscles unlikely in spite of using surface electrodes even though this cannot be completely ruled out. Of note, surface recordings are susceptible to contraction‐related changes in muscle–electrode spatial configuration. As the diaphragm descends and flattens during inspiration, electrode–muscle distance should increase, which, if anything, should increase latency rather than decreasing it as was observed in our participants.

In conclusion, our findings confirm that the diaphragm exhibits task‐dependent corticospinal modulation governed by principles similar to those described in other skeletal muscles, with both static and dynamic inspiratory effort facilitating the response to cortical inputs. However, such modulation appears contingent on rapid execution, as slow dynamic commands fail to produce facilitation patterns more marked than those elicited by static commands. This discrepancy with known data in hand muscles possibly reflects the hybrid automatic–voluntary control of respiratory muscles: it can thus be hypothesized that the constant influence of the bulbospinal input to respiratory motoneurons suffices to make them less sensitive to slow cortical inputs. Of note, fast voluntary inspirations not only increase the diaphragm response to TMS but also to accelerate it, another difference with published hand muscle data. This would be consistent with the capacity of the cortical control of breathing to transiently override automatic respiratory drive under high metabolic demand in health (e.g., intentional coordination of breathing with locomotion, speech during intense exercise, or controlled breathing during swimming) (as discussed in Lavin et al. (2015), Rotstein et al. (2004), and Takano & Deguchi (1997)) or in disease (e.g., preserved capacity of patients with severe respiratory diseases to speak or perform pulmonary function tests).

## AUTHOR CONTRIBUTIONS

**Alexandre Demoule:** Conceptualization; data curation; formal analysis; investigation; methodology. **Eric Verin:** Data curation; investigation. **Capucine Morélot‐Panzini:** Data curation; investigation. **Christian Straus:** Conceptualization; data curation; investigation; methodology. **Thomas Similowski:** Conceptualization; data curation; formal analysis; investigation; methodology; project administration; supervision.

## FUNDING INFORMATION

This study was funded in part by a “contrat triennal ‘Legs Poix’ de la Chancellerie de l'Université de Paris”, Paris, France, and by the Association pour le Développement et l'Organisation de la Recherche en Pneumologie (ADOREP), Paris, France. None of the funding sources intervened in study design, data acquisition and management, or the writing of the manuscript.

## CONFLICT OF INTEREST STATEMENT

AD has nothing to disclose regarding the present study. Outside this study, he reports grants from the French Ministry of Health, Respinor, Liberate Medical, Bio Aegis/Ergomed, consulting fees from Liberate Medical, Respinor, Black Fur Medical, WK Health Medical Research, support for attending meetings from Respinor. EV has nothing to disclose regarding the present study, and nothing to disclose outside it. CMP has nothing to disclose regarding the present study. Outside this study, she reports over the last 3 years research grant from Chiesi France, personal fees for consulting and teaching activities from Chiesi France, Sanofi France, Menarini France, GSK France, Oxylis, SOS Oxygène, ResMed France, Fisher and Paykel, Breas, and Lowenstein Medical France, support for attending meeting from ASV, Asten, SOS Oxygène. CS has nothing to disclose regarding the present study, and nothing to disclose outside it. TS has nothing to disclose regarding the present study. Outside this study, he reports over the last 3 years personal fees for consulting and teaching activities from Chiesi France, OSO‐AI, and Lowenstein Medical France. He is a stock shareholder of startup Austral Dx. He is listed as inventor on several issued or pending patents in the field of respiratory physiology, respiratory neurophysiology, and respiratory care.

## ETHICS STATEMENT

The study was conducted in accordance with the Declaration of Helsinki and was approved by the appropriate ethical and regulatory body (Comité de Protection des Personnes Île‐de‐France VI).

## CONSENT

All participants provided written informed consent.

## GENERATIVE ARTIFICIAL INTELLIGENCE

No part of this manuscript has been produced using generative artificial intelligence.

## Data Availability

The complete data set used in this study will be made available to researchers upon reasonable request addressed to the corresponding author.
